# Availability of Arg, but Not tRNA, Is a Rate-Limiting Factor for Intracellular Arginylation

**DOI:** 10.3390/ijms23010314

**Published:** 2021-12-28

**Authors:** Irem Avcilar-Kucukgoze, Brittany MacTaggart, Anna Kashina

**Affiliations:** School of Veterinary Medicine, University of Pennsylvania, Philadelphia, PA 19104, USA; iavcilar@vet.upenn.edu (I.A.-K.); britmact@pennmedicine.upenn.edu (B.M.)

**Keywords:** arginylation, arginyltrasferase, tRNA, arginine, arginine metabolism

## Abstract

Protein arginylation, mediated by arginyltransferase ATE1, is a posttranslational modification of emerging biological importance that consists of transfer of the amino acid Arg from tRNA to protein and peptide targets. ATE1 can bind tRNA and exhibits specificity toward particular tRNA types, but its dependence on the availability of the major components of the arginylation reaction has never been explored. Here we investigated key intracellular factors that can potentially regulate arginylation in vivo, including several tRNA types that show strong binding to ATE1, as well as availability of free Arg, in an attempt to identify intracellular rate limiting steps for this enzyme. Our results demonstrate that, while modulation of tRNA levels in cells does not lead to any changes in intracellular arginylation efficiency, availability of free Arg is a potentially rate-limiting factor that facilitates arginylation if added to the cultured cells. Our results broadly outline global pathways that may be involved in the regulation of arginylation in vivo.

## 1. Introduction

Protein arginylation, mediated by arginyltransferase ATE1, is a posttranslational modification of emerging biological importance that consists of transfer of the amino acid Arg from tRNA to protein and peptide targets. ATE1 typically targets N-terminally exposed acidic amino acid residues, Asp and Glu, but has also been recently found to transfer Arg to the acidic Asp and Glu side chains, suggesting that this enzyme can mediate two types of Arg linkages [1], likely dependent on the sequence context [2] and possibly modulated by in vivo binding partners. A number of in vivo arginylation substrates have been identified [1,3,4,5,6,7], including components of the actin cytoskeleton [3,7], nucleotide biosynthesis [8], and other major pathways [6].

ATE1 is expressed from the single *Ate1* gene in animals and fungi [9], and two genes, *Ate1* and *Ate2*, in plants, where these enzymes are believed to be functionally redundant [10]. In mammals, ATE1 is represented by four alternatively spliced isoforms that share very high sequence identity [11], even though they exhibit differences in their target site specificity [2,11,12].

ATE1 utilizes Arg-charged tRNA^Arg^ as the donor of the arginyl group. Thus, arginylation reaction in vivo relies on the availability of free Arg and tRNA^Arg^, as well as on the activity of Arg–tRNA synthetase (RARS) that generates Arg–tRNA^Arg^ for translation as well as arginylation. Thus, arginylation in vivo exists in an interplay with translation machinery as the availability of all these reaction components. ATE1 can arginylate its targets independently of the presence of ATP [12] and can utilize Arg-charged tRNA-derived fragments as donors of Arg [13]. No dedicated ATE1 interaction partners have been identified to date, making it unclear of how this ubiquitous enzyme is regulated in vivo.

ATE1 can bind directly to tRNA and exhibits specificity toward particular tRNA types [13]. Based on the repertoire of the tRNA that is either over- or underrepresented in ATE1 preparations, our lab previously proposed a hypothesis that these tRNA species can differentially modulate ATE1’s in vivo activity, acting as arginylation inhibitors in vivo, but this possibility has never been explored.

Here we investigated key intracellular factors that can potentially regulate arginylation in vivo, including several tRNA types that show strong binding to ATE1, as well as availability of free Arg, in an attempt to identify intracellular rate limiting steps for this enzyme. Our results demonstrate that, while modulation of tRNA levels in cells does not lead to any changes in intracellular arginylation efficiency, the availability of free Arg is a potentially rate-limiting factor that facilitates arginylation if added to the cultured cells. Our results broadly outline global pathways that may be involved in the regulation of arginylation in vivo.

## 2. Results

### 2.1. Small RNAs, including tRNAs, Can Specifically Inhibit Arginylation In Vitro

**Figure 1:**
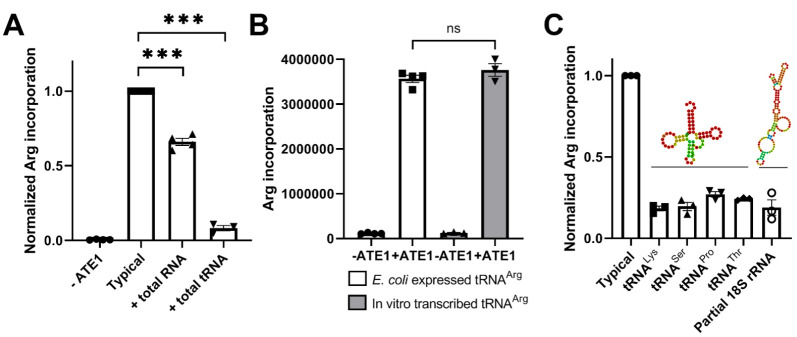
tRNA and 18S rRNA inhibit arginylation in vitro. (**A**) Incorporation of ^3^H-Arg into angiotensin II in a typical arginylation reaction (typical), with and without addition of different RNA preparations, as marked on the figure. (**B**) Comparison of native tRNA^Arg^ expressed in *E. coli* and in vitro transcribed tRNA^Arg^ in the in vitro arginylation reaction. (**C**) Incorporation of ^3^H-Arg into angiotensin II in a typical arginylation reaction (typical), with and without addition of different tRNA types, as marked on the figure. Structural models of a typical tRNA (modeled from mouse tRNA^Thr^ACG-1-1) and the 18S rRNA fragment used in the reaction are shown on top for comparison of the predicted stem-loop structures. Cpm counts in each reaction set were normalized to the typical reaction. Error bars represent SEM, at least n = 3 independent reactions; *** *p* < 0.001, ns, not significant, Welch’s *t*-test.

We previously found that ATE1 specifically interacts with tRNAs and exhibits preferences toward specific non-Arg tRNA subtypes [13]. Since none of these tRNA species is utilized for arginylation, we hypothesized that they may act as competitive inhibitors of arginylation and/or play another role in modulating arginylation. To test this, we performed in vitro arginylation of angiotensin II, a peptide shown in our previous studies to constitute a highly efficient arginylation substrate [1], in the presence of different RNA species (Figure 1). The addition of total RNA inhibited a typical arginylation reaction by approximately 40%. In contrast, the addition of the same amount of total tRNA inhibited the arginylation reaction by >90%. Thus, total tRNA inhibits arginylation much more efficiently than total RNA, suggesting that tRNA has a specific inhibitory effect on arginylation.

In our previous study, different tRNA species exhibited prominent differential binding to ATE1 [2]. Moreover, tRNA^Pro^, tRNA^Lys^, and tRNA^Thr^ were strongly enriched in ATE1 preparations, while some of the other tRNAs, e.g., tRNA^Ser^, were prominently underrepresented, leading to a hypothesis that these strongly bound non-Arg tRNAs may act as specific inhibitors of arginylation. To test whether these specific tRNA types can have selective effects on ATE1-mediated arginylation reaction, we performed arginylation of angiotensin II in the presence of in vitro transcribed tRNA^Pro^, tRNA^Lys^, tRNA^Ser^, and tRNA^Thr^. We also included a partial transcript corresponding to a 131 nt fragment of 18S ribosomal RNA, since ATE1 has previously been proposed to interact with the ribosomes [12]. Notably, all of these tRNA types, as well as 18S rRNA fragment, had similar inhibitory effects on arginylation. Thus, contrary to our prior hypothesis, inhibition of arginylation reaction by small RNA does not appear to exhibit strong specificity toward specific types of tRNA, and it can also occur in the presence of short rRNA.

### 2.2. Increased tRNA Levels Do Not Affect Arginylation Efficiency In Vivo

**Figure 2:**
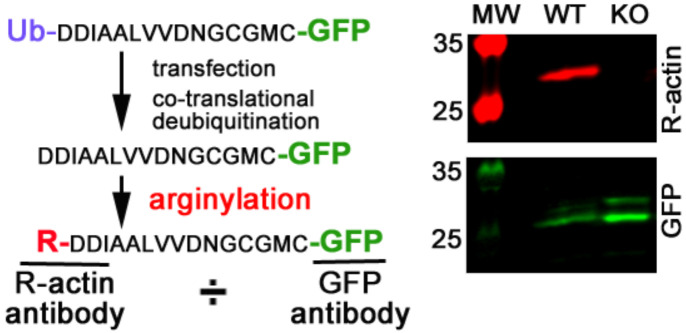
Arginylation sensor for detection of intracellular arginylation. Left, diagram; right, representative Western blot of the arginylation sensor transfected into wild-type (WT) and *Ate1* knockout (KO) mouse embryonic fibroblasts and visualized with antibodies to arginylated β actin (R-actin) and GFP. Ratio of the R-actin:GFP signal was used in the intracellular assays described below.

Next, we tested whether the modulation of intracellular tRNA levels affects arginylation efficiency in vivo. To do this, we used an intracellular arginylation sensor that was previously described in Reference [14]. This sensor consists of an expression construct, in which an N-terminal ubiquitin moiety is followed by the N-terminal 15 residues of β actin, a known target for N-terminal arginylation [7], with C-terminally fused GFP. The ubiquitin moiety is efficiently co-translationally removed by deubiquitinating enzymes, exposing the N-terminal Asp residue of the actin-derived sequence that serves as a target for arginylation. Arginylation of this fusion protein can then be detected by using arginylated actin antibodies [15], and the arginylation efficiency can be quantified as a ratio of arginylated actin signal to the signal of GFP (Figure 2 and Reference [14]).

**Figure 3:**
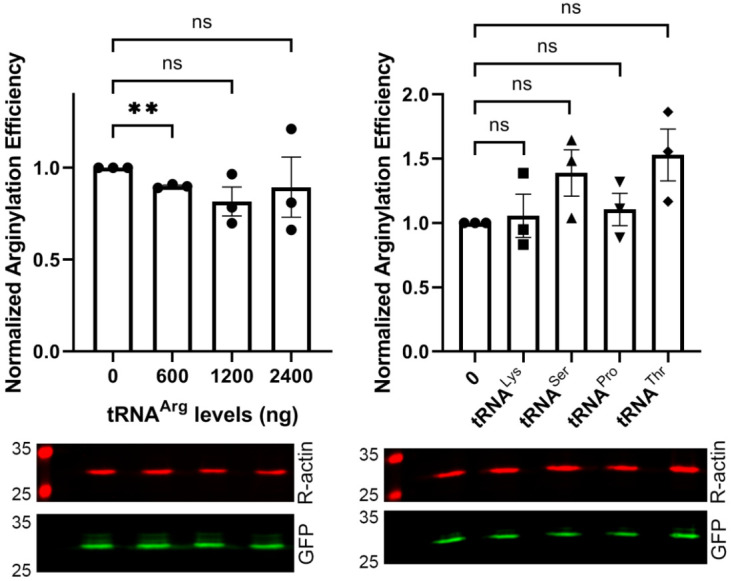
Transfection of cells with different tRNA species does not significantly affect intracellular arginylation. Charts (**top**) and representative Western blot images (**bottom**) of sensor-based quantification of arginylation in cells transfected with different amounts of tRNA^Arg^ (**left**) or 1200 ng of different tRNA species as indicated (**right**). Error bars represent SEM, n = 3 independent transfections; ** *p* < 0.01; ns, not significant; Welch’s *t*-test.

We used this detection system to test whether transfection of cells with increasing levels of different types of exogenous tRNA can affect the efficiency of arginylation of the sensor simultaneously co-transfected into the same cells. In these experiments, we used direct transfection of in vitro transcribed tRNA into cells, a method that enables precise control over the added tRNA levels and has been previously shown to be highly effective in introducing active translation-competent tRNA into cells [16]. To test if increasing the levels of tRNA^Arg^ increases the level of arginylation, we transfected cells with increasing amounts of tRNA^Arg^ (Figure 3, left). However, we found no increase in arginylation after this transfection. Thus, tRNA^Arg^ availability is not a limiting factor for intracellular arginylation.

Next, we tested whether an increase in the intracellular levels of the tRNA found to be inhibitory in our in vitro studies (Figure 1) has an effect on arginylation in vivo. In these experiments, transfection of tRNA^Pro^, tRNA^Lys^, tRNA^Ser^, and tRNA^Thr^ did not affect the overall arginylation efficiency (Figure 3, right).

Thus, increased levels of intracellular tRNA do not exert any activatory or inhibitory effects on arginylation.

### 2.3. Increased Levels of Arg in the Media Facilitate Arginylation

**Figure 4:**
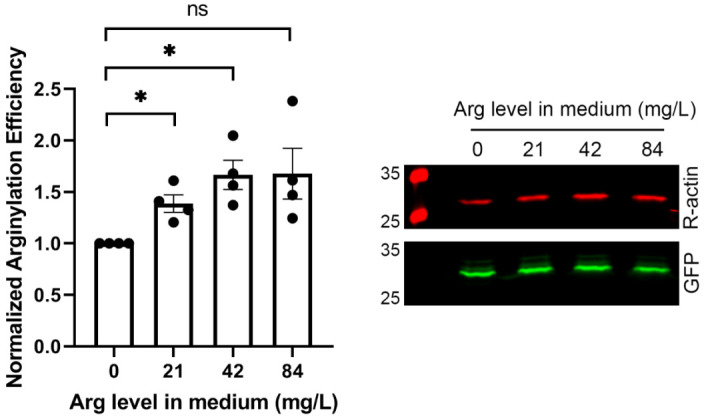
Addition of free Arg to the tissue culture medium increases the efficiency of arginylation. Chart (**left**) and representative Western blot image (**right**) of sensor-based quantification of arginylation in cells grown in Arg-free media supplemented with different amounts of Arg, as indicated. Error bars represent SEM; n = 4 independent transfections; * *p* < 0.05, ns, not significant, Welch’s *t*-test.

In search of other intracellular factors that could potentially be limiting in the arginylation reaction, we tested the effect of different concentrations of free Arg added into the cell culture media. To ensure that the media remained Arg-free prior to the Arg addition, we used dialyzed serum and DMEM media without L-arginine for this experiment. We then tested arginylation efficiency at increasing Arg concentrations, using the intracellular Arg sensor (Figure 4).

**Figure 5:**
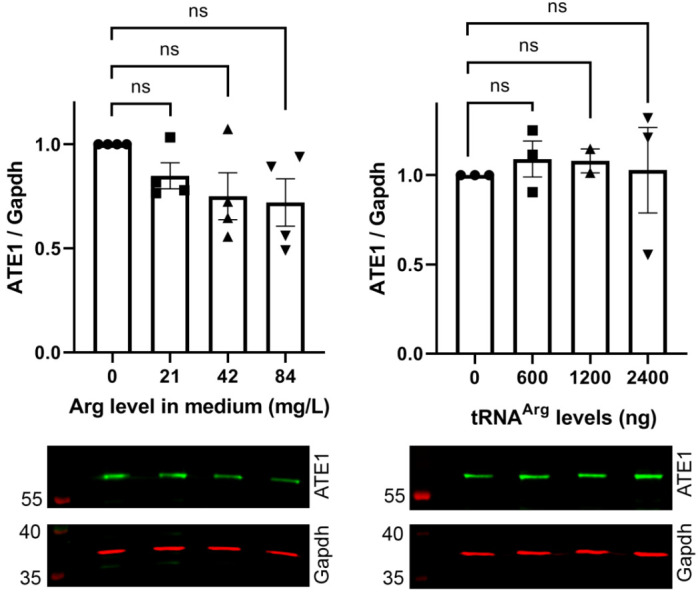
Addition of free Arg or tRNA^Arg^ to cells does not affect the levels of ATE1. Charts (**top**) and representative Western blot images (**bottom**) of sensor-based quantification of arginylation in cells grown in media supplemented with different amounts of Arg (**left**) or transfected with different amounts of tRNA^Arg^ (**right**). Error bars represent SEM, at least n = 3 independent transfections; ns, not significant, Welch’s *t*-test.

Arginylation efficiency increased nearly linearly from 0 to 42 mg/L, a concentration similar to that in typical culture media formulations. Furthermore, doubling the amount of Arg in the media did not facilitate an additional increase in arginylation, suggesting that this Arg concentration is sufficient to support intracellular Arg availability for arginylation. As a control experiment, we compared the levels of ATE1 in cells treated with different Arg concentrations or transfected with different amounts of tRNA^Arg^. None of these treatments changed intracellular ATE1 levels (Figure 5). Thus, the arginylation increase seen upon the addition of Arg to the culture media directly reflects an increase in the efficiency of the arginylation reaction.

Together, these results suggest that availability of Arg, but not tRNA^Arg^, constitutes a limiting factor that regulates the efficiency of arginylation in vivo.

## 3. Discussion

Our results constitute a global approach to understanding the limiting factor(s) that may contribute to regulation of arginylation in vivo by regulating the availability of the key components of the arginylation reaction, as well as potential modulation of ATE1 activity or substrate interaction via direct binding to tRNA. We find that the addition of different tRNA species greatly inhibits arginylation in vitro. Since ATE1 has been previously found in our studies to be highly specific to Arg-conjugated tRNA^Arg^, this inhibitory effect is unlikely to occur via impairment of ATE1’s enzymatic functions, and it may potentially occur via direct competition between small stem-loop tRNAs and tRNA^Arg^ for ATE1 binding. Notably, none of the tRNAs previously shown to interact with ATE1, including tRNA^Arg^, facilitates or inhibits arginylation in vivo. Thus, intracellular tRNAs are unlikely to play a major role in binding ATE1 in vivo, at least not in a way that affects its activity in arginylation. Moreover, the availability of tRNA^Arg^ does not appear to be a limiting step for in vivo arginylation. It is possible that, in contrast to in vitro, the in vivo balance between the arginylation reaction components is precisely regulated, so that the inhibitory effects of small RNAs seen in vitro do not manifest themselves in the in vivo context or occur only locally, without altering the global arginylation levels.

**Figure 6:**
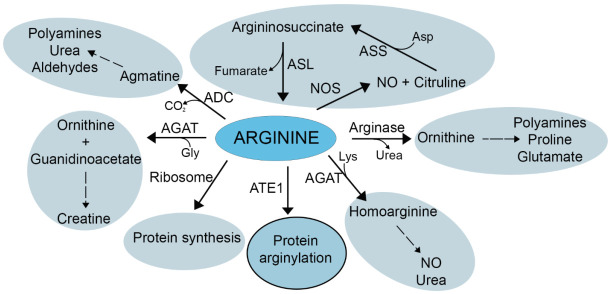
Arginine is a central component in a number of key physiological pathways. Major pathways that critically depend on Arg levels in the cell are shown. Alterations in Arg availability has the potential to critically affect the balance of these pathways, with important consequences to the cell.

Our results suggest that the availability of free Arg may be one of the factors that can balance arginylation with other intracellular pathways that include Arg (Figure 6). At a first glance, this result does not appear that surprising, given that Arg is one of the two known essential components of the arginylation reaction. However, the fact that Arg metabolism in vivo is very complex and Arg availability can serve as a key factor and a rate-limiting step in several major metabolic pathways (Figure 6) puts this result into a different light. Given this complexity, it appears possible that local modulation of Arg levels and its flux into different pathways in vivo may potentially also serve as a finer regulator of specific arginylation events. This possibility requires further studies.

Even though increasing tRNA levels in cultured cells does not affect arginylation, strong and specific inhibition of arginylation in vitro upon addition of tRNA suggests that tRNAs, and possibly other small RNAs, have the potential to exert effects on ATE1 that may not translate into global arginylation changes but could still act at the local levels. In this case, it appears likely that these tRNAs act as direct or indirect competitors that could prevent binding of tRNA^Arg^ and thus inhibit arginylation. Given some similarities between the stem-loop structures of tRNA and the 18S rRNA fragment used in our study (Figure 1), it is possible that such stem-loop structures in general facilitate ATE1–RNA binding. In support, total RNA preparations have a far less inhibitory effect on in vitro arginylation (Figure 1), potentially because the longer mRNA that constitute the majority of these preparations are less prone to tight stem-loop formation. Elucidating the ATE1 structure will shed light onto this question and improve our understanding of ATE1–RNA interaction and its specificity.

In addition to tRNA and Arg, other factors that were not tested here likely play a role in the regulation of arginylation. Since ATE1 utilizes Arg–tRNA^Arg^, the activity of RARS is also important, since this enzyme regulates the availability of Arg–tRNA^Arg^ for both translation and arginylation. The role of RARS and other potential factors modulating arginylation in vivo constitute an exciting direction of future studies.

## 4. Materials and Methods

### 4.1. tRNA Preparation and In Vitro Arginylation Reaction

Mouse tRNA^Thr^ CGT 1-1, tRNA^Ser^ AGA 1-1, tRNA^Lys^ CTT 3-1, tRNA^Pro^ AGG 1-1, and partial 18S rRNA (131 nt) were in vitro transcribed by MEGAshortscript T7 Transcription Kit, according to the manufacturer’s protocol. The RNA transcripts were renatured by heating up at 95 °C for 2 min, followed by incubation at RT for 3 min and further at 37 °C for 5 min. Total RNA was extracted from mouse liver by TRIZOL, according to the manufacturer’s protocol. Total tRNA was extracted by ZR small-RNA PAGE recovery kit. *E. coli*–expressed tRNA^Arg^ was performed as previously described [17]. All tRNAs purified from native sources were deacylated as the final step prior to addition to the arginylation reaction.

The in vitro arginylation reaction was performed in a 50 µL volume containing 50 mM HEPES, pH 7.5, 25 mM KCl, 15 mM MgCl_2_, 0.1 mM DTT, 2 mM ATP, 5 µM [^3^H]-Arginine, 15 µM angiotensin II, 5 µM tRNA^Arg^, 1 µM RARS, and 0.5 µM ATE1. To this reaction, 10 µg of total RNA/total tRNA was added. As shown in Figure 1C, the in vitro arginylation reaction was performed in 25 µL volume, and 2 µg of each tRNA (3.4 µM tRNA/1.88 µM 18S rRNA fragment) was added separately. After mixing, the reaction was incubated at 37 °C for 5 min and then heated at 95 °C for 15 min. Angiotensin II peptide was purified by using C18 spin columns and analyzed on a scintillation counter (Beckman Coulter LS 6500) to measure Arg incorporation.

### 4.2. Cell Culture, tRNA Transfection, and Arg Addition

HEK293T and MEF cells were grown in Dulbecco’s modified Eagle’s medium, with GlutaMAX^TM^ supplement (DMEM + GlutaMAX, Gibco) with 10% fetal bovine serum and 1% Penicillin–Streptomycin at 37 °C, with 5% CO_2_. Then tRNA transfections were performed in 6-well plates. In vitro transcribed tRNA was mixed with the 4 µg of Arg sensor plasmid, and this mixture was directly added to the cells in combination with Lipofectamine 2000. For Figure 3, right, 1200 ng tRNA was transfected. For Figure 4, we used dialyzed serum (A33820-01, Gibco) and DMEM media deficient in both L-lysine and L-arginine (88364, Thermo Scientific). L-lysine was added to make the 146 mg/L final concentration. After 48 h, the transfected cells were washed once with phosphate-buffered saline (PBS, Corning) and harvested by scraping and centrifugation. Cell pellets were lysed in 4 × SDS Sample Buffer at the w:v ratio of 1:20 (1 mg cell pellet: 20 µL buffer), followed by boiling the samples for 10 min. Then 10 µL of each sample was loaded for SDS–PAGE electrophoresis.

### 4.3. Western Blotting and Quantification of Arginylation

The gels were transferred to nitrocellulose membrane at 100 V for 60 min. The blots were then blocked by 5% milk in PBST at 4 °C for 16 h and incubated with primary antibodies of rabbit anti-R-actin (ABT264 EMD Millipore, 1:2000), mouse anti-GFP (Ab1218 Abcam, 1:3000), rat anti-ATE1 (homemade, 1:1000), or mouse anti-Gapdh (Ab8245 Abcam, 1:5000) for 60 min at room temperature. Secondary antibodies (1:5000) conjugated to IRDye800 or IRDye680 were used, and images were acquired by using Odyssey Imaging System. Images were analyzed by using Image Studio Lite. Ratio of R-actin signal (red channel) and GFP antibody signal (green channel) was used as a measure of arginylation.

## Data Availability

All supporting data is presented in the manuscript.
